# The Perspectives of Diabetic Men about Sexual Problems, Consequences, and Therapeutic Measures

**DOI:** 10.21315/mjms2018.25.2.14

**Published:** 2018-04-27

**Authors:** Ali Zakiei, Behrooz Faridmarandi, Saeid Komasi

**Affiliations:** 1 Social Development and Health Promotion Research Center, Kermanshah University of Medical Sciences, Kermanshah, Iran; 2 Psychology Department, Islamic Azad University, Kermanshah, Iran; 3 Clinical Research Development Center, Imam Reza Hospital, Kermanshah University of Medical Sciences, Kermanshah, Iran

Dear Editor,

Sexual dysfunction in diabetic men is one of the common outcomes of the disease (1), thereby leading to loss of libido, impaired ejaculation and erection, general sexual dysfunction, and reduced sexual satisfaction (2). Despite the common side-effects in diabetic patients, it appears that the attention of health care professionals is generally directed to diabetes control, and the marginal problems of these patients, such as sexual health, are less considered. A review of the literature revealed that most previous studies (3) focused on women with diabetes and only few studies have been conducted on men.

Based on these considerations, the present study aimed to assess the viewpoints of diabetic men in relation to sexual problems, consequences, and therapeutic measures. A total of 51 diabetic male patients were evaluated. The mean age of participants was 53.4 ± 9.3 years. In terms of education level, 12.1% of them were illiterate, 32.7% had an elementary education, and 46.2% had secondary or university education. Moreover, the employed patients accounted for 58.9% of the population under study, while 3.7% and 37.3% were unemployed and retired, respectively. The results also demonstrated that the mean duration of diabetes was 9.14 ± 4.15 years.

In the phenomenological-qualitative study, 51 patients were studied, and the inclusion criteria included visitors diagnosed with type 2 diabetes (at least two years should have elapsed from the time of diagnosis), visiting the Kermanshah Diabetes Research Center. It should be noted that the data were collected using a semi-structured interview. Based on the results, 45 patients (more than 88%) reported that their sexual function was affected by diabetes since the onset of the disease. “I have not had a pleasant intercourse for about three years,” said a 62-year-old patient with a history of five years of the disease. These patients were asked whether the decrease in their sexual function affected the quality of their marital life. They answered that their lives were seriously affected. “Because of my impotence, my wife asked for a divorce and separated, and life is hard for me,” said, a 60-year-old patient (with a history of six years of illness).

The patients were then asked whether they had taken any measures to resolve their sexual problems. The answers to this question could be divided into three categories. The first group, which included about 60% of the patients, stated that they had not taken any action. The second group, which contained 30% of the patients, stated that they would return to specialist doctors. “I’ve had a lot of problems before, but I’ve been cured by a doctor since I started the prescription last year,” said a 59-year-old patient (with a history of 15 years of illness). But some believed that medication did not have any effects. For example, a 60-year-old patient (with a history of 10 years of illness) said “the doctor prescribed me medicine but I did not take it because it troubles my heart*.*” However, the third category (which included 10% of the patients) stated that they were turning to opioids to solve their problems. A 62-year-old patient said, “If I take opioids, I will have a happy intercourse*.*”

The patients were also asked whether the sexual problems had caused them to feel that life was meaningless. The answer that most of them gave was negative and they said that life was meaningful with their children. A 62-year-old patient (with a history of seven years of illness) said “the greatest meaning of my life is my children*.*” However, some had different opinions. For example, a 65-year-old patient (with a history of two years of illness) cried after hearing the question and said “the disease has caused me a lot of troubles and is more difficult than any other illness, and I feel that my life has become meaningless*.*”

Copeland et al. argued that interest, satisfaction, and ability to engage in sexual activities were seriously affected by diabetes. Besides, sexual functioning may be negatively affected by medications or other interventions that are used to monitor or treat this chronic illness (4). In other words, one of the reasons for the low level of sexual functioning in diabetic patients is the effect of the medications used for this disease. Also, to explain the results in this section, it can be noted that diabetes, vascular and neurological disorders, and psychological problems are among the main causes for the development or intensification of sexual dysfunction (5).

Therefore, in general, it can be argued that the sexual satisfaction and sexual functioning of patients with diabetes are undesirable. Given the increasing prevalence of type 2 diabetes, this issue should be taken seriously and in addition to the treatment and control of the disease, attention should be given to the sexual satisfaction and sexual functioning of these patients, and necessary interventions should be taken into consideration along with the routine treatments for sexual functioning and sexual satisfaction. Moreover, in addition to prescribing medication, it should be noted that psychological problems play a key role in this regard. For example, a 55-year-old patient with a history of six years of illness said “I’m taking the medicines prescribed by the doctor, but they do not work*.*” This issue was due to life problems and it was found in the interview that two of his family members were suffering from post-traumatic stress disorder (PTSD). Accordingly, it is recommended that besides treating the disease, these people should be advised by psychologists to solve their problems.
